# Analyzing psychological mechanism of physical activity enhancing Chinese L2 learning efficacy with inhibiting aggressive behavior using cross-lagged panel model

**DOI:** 10.1371/journal.pone.0324984

**Published:** 2025-06-03

**Authors:** Zixia Bu, Ziqi Gao, Yu Liu, Haohan Yu, Jiyuan Li, Fuqiang Dong, Shan Jiang

**Affiliations:** 1 School of lnternational Chinese Language Education, Beijing Normal University, Beijing, China; 2 College of Physical Education and Sports, Beijing Normal University, Beijing, China; 3 Division of Sports Science and Physical Education, Tsinghua University, Beijing, China; 4 School of Marxism, Beijing Jiaotong University, Beijing, China; 5 College of Physical Education, Minzu University of China, Beijing, China; 6 Department of Sports Science and Physical Education, The Chinese University of Hong Kong, Shatin, Hong Kong, China; University of Tartu, ESTONIA

## Abstract

**Objective:**

For international students coming to China, the process of learning Chinese is very difficult. At the same time, chronic academic underachievement can lead to aggressive behavior (AB). Although physical activity (PA) has been shown to improve learning performance and psychological well-being, its effectiveness in Chinese language learning and AB has not been clarified. Therefore, the present study examined PA as the main variable and explored its dosage profile in Chinese learning and alleviation of AB.

**Methods:**

A total of 964 international students from different countries from 8 universities in Beijing were selected as the study population and were assessed using the International Physical Activity Questionnaire (IPAQ), Buss-Perry Aggression Questionnaire (BPAQ) and Hanyu Shuiping Kaoshi (HSK) for 3 longitudinal follow-ups. Analyses of variance, correlations and path models were performed using ANOVA, Pearson and cross-lagged panel model (CLPM).

**Results:**

High PA level accounted for 18%, moderate PA level for 35%, and light PA level for 47%. PA was significantly elevated on AB and all sub-indicators except HSK (F = 1.58–4.38, η² = 0.03–0.05, P < 0.05), and was decreasingly related to physical aggression, verbal aggression, and angry (F = 4.38, η² = 0.03,; F = 3.24, η² = 0.04,; F = 2.37, η² = 0.04; P < 0.01). In terms of correlation, cross-sectional comparison showed that AB was significantly negatively correlated with both HSK and PA (r = −0.41, P < 0.01; 1 = −0.44, P < 0.01). The longitudinal results showed a decreasing trend of negative correlation between AB and PA under T1-T3 stages (r = −0.29, r = −0.44, P < 0.01), and no significant change in the degree of negative correlation with HSK (r = −0.28, r = −0.35, P < 0.01). The positive correlation between HSK and PA did not change significantly (r = 0.15, r = 0.18, P < 0.01). The positive correlation of PA itself decreased over time (r = 0.83, r = 0.76, P < 0.01). The CLPM results show that under T1 phase, PA negatively affects AB in T1 and T2 phases (β = −0.42, β = −0.18) and positively affects HSK in T2 phase (β = 0.24). AB negatively affects T1 and T2 phase HSK (β = −0.25, β = −0.26). HSK negatively affects AB in T2 phase (β = −0.11). Under T2 phase, PA negatively affects T3 phase AB (β = −0.07) and positively affects T3 phase HSK (β = 0.22). AB negatively affects T3 stage HSK (β = −0.10). HSK negatively influences T3 stage AB (β = −0.08).

**Conclusions:**

(1) PA may have beneficial effects on both Chinese L2 learning efficacy and the reduction of AB. (2) Higher levels of PA are likely to strengthen these effects. (3) Establishing daily PA habits may serve as an effective strategy to enhance L2 learning outcomes while simultaneously reducing AB in learners.

## Introdvuction

Numerous studies have shown that physical activity (PA) has a significant positive impact on cognitive function, particularly in the areas of executive function, learning and memory [1]. In the Mental Health Gap Action Programme (mhGAP) 2023, World Health Organization (WHO) has shown that physical activity (PA), as a non-pharmacological intervention, can be effective in improving an individual’s cognitive and executive functioning and academic performance [2]. From a mechanistic perspective, PA may optimize cognitive control systems by enhancing the individual’s neuroplasticity and intracranial transmitter release, promoting neurogenesis and modulating cerebral blood flow (CBF), and improving prefrontal cortex and hippocampal function related to learning and memory behaviors [3]. This set of mechanisms involves important functions related to language learning. Regular PA has been shown to be effective in improving individual language learning efficacy and mood [4]. This is because language acquisition is highly dependent on higher cognitive processes such as working memory, semantic processing and attentional modulation, and the neurophysiological underpinnings enhanced by PA can directly support an individual’s efficiency in the phonological perception, lexical memorization and grammatical processing segments of the language learning process [5]. This is especially true for students and adolescents [6]. Therefore, there is a clear scientific value and application of PA as an intervention in the design of language teaching and learning.

On the other hand, as China’s international influence grows and the process of globalization and regionalization deepens, the Belt and Road Initiative in particular has received great attention and recognition from the international community. As a result, the scope of Chinese language use and broadcasting is also expanding [7]. According to the China Student Aid Development Report 2017, the number of international students coming to China has increased to 443,000 since 2016 [8]. In the process of international students studying in China, Chinese language as one of the compulsory courses occupies more than one sixth of the time in all courses for international students [9]. Although the proportion of Chinese courses is very large, its glyphs, rhythms and composition are more complicated than English, thus causing much difficulty for Chinese learners, and it was once even called ‘Devil’s Language’ [10]. The problems associated with the language barrier are significant. The frustration-aggression theory suggests that individuals who are academically frustrated are more likely to develop psychological problems [11]. One of the most prominent predisposing conditions is aggressive behavior (AB) [12,13]. A multinational study found that people with better academic performance were 20% to 35% less likely to develop AB [14]. For the group of international students coming to China, issues such as language learning barriers or communication barriers can easily have a negative impact on individual mental health [15]. Therefore, how to help international students maintain their mental health while effectively promoting Chinese language learning has become one of the hot topics in Chinese language research and language psychology.

Although previous evidence has found the facilitative effects of PA on language learning, as the science of exercise grows, studies continue to find that different levels of PA have different effects on improving second language learning. For example, Tarkington found that adolescents with regular PA habits had lower levels of negative affect and academic difficulties than a blank control group. This effect was found to increase gradually with the amount of exercise [16]. Similarly, by constructing a CHAID decision tree, Du found that there is a threshold for the positive contribution of PA to academic effectiveness. Specifically, students with 16–25 minutes of PA at least once a week outperformed about 88% of other students in terms of GPA and foreign language learning [17]. So far, although the improvement effect of PA on second language learning efficacy has been confirmed, there is still insufficient evidence to prove whether it is applicable to Chinese language learning. Therefore, in order to investigate whether PA can effectively reduce the probability of problematic behavior such as AB in the process of improving Chinese second language learning efficacy, the present study constructed a cross-lagged panel model (CLPM) with PA as the main variable, and analyzed the psychological pathway model and the brain structure basis of the three. This study provides more scientific exercise prescription and suggestions for international students and language lovers to learn Chinese.

Currently, there is a relatively limited amount of literature exploring the contribution of PA to second language learning outcomes. After a systematic search and review of the field, this study found that Gao’s and Bu’s studies were the most similar. Among them, Bu grouped all participants according to their PA level in a cross-sectional survey and analyzed the differences in the effects of PA level on international students’ Chinese language learning [18]. Gao, on the other hand, also used the effect of second language learning as an outcome indicator, but in a different way, using enjoyment of foreign languages as the main dependent variable and constructing a CLPM based on three longitudinal follow-ups. This provided a more rigorous quantification of the impact of second language learning [19]. The present study, however, will build on these two studies by taking PA as the main variable, Chinese learning effect as the dependent variable, and AB as the mediator variable, conducting three follow-up visits spanning several months, and establishing the CLPM. The purpose is to quantify the specific facilitating effect of PA on Chinese learning effect in a more rigorous and scientific manner. The present study, based on the first two, proposed the following hypotheses: (I) There is an ameliorative effect of PA on AB and Chinese learning, and this effect may be positively related to intensity. (II) PA, as the main variable, may be a negative predictor of AB and a positive predictor of Chinese learning.

## Materials and methods

### Participants

This study started on 10 September 2022 and ended in June 2023. Using the cluster random sampling method, 964 international students from different countries from 8 universities in China were initially selected as the subjects of the study, taking the international student classes in each university as the unit. The inclusion criteria for the subjects were: (1) Current exchange or international students, including undergraduate, master’s, and doctoral students whose educational institution is a senior college or university. (2) They were conscious and able to complete the questionnaire without difficulty and had no history of mental retardation or mental illness. (3) Subjects volunteered to participate in this study and did not refuse to complete the survey. (4) Informed consent for the survey was given by the counselor (classroom teacher), and the questionnaire was completed after signing it. The procedures were conducted in accordance with the National Institutes of Health Guidelines and were approved by the Ethics Committee of Human Experimentation of Beijing Normal University (Authorization no. TY20220905). In addition, all participants have signed an electronic version of the informed consent form via online collection, and a participant representative will sign the paper informed consent form again before the experiment. All participant information were protected by research staffs. A statement to confirm that all approaches were carried out in accordance with relevant guidelines and regulations. Informed consent forms were obtained from all subjects. Participant demographics are detailed in Table 1.

**Table 1:** Demographic information of participants.

Parameter		All	Level of physical activity		
			Light	Medium	High
Gender (n)	Male	653	281	248	124
	Female	311	152	104	55
Nationality source (n)	Asia	587	328	154	105
	Europe	115	29	52	34
	America	58	16	27	15
	Africa	161	84	45	32
	Oceania	43	19	13	11
Education level (n)	Undergraduate	693	279	223	191
	Postgraduate	241	119	68	54
	Doctor	30	17	9	4
Type of specialization (n)	Science student	561	247	173	141
	Humanities student	403	153	138	112
Age (yr)		21.89 ± 1.86	22.64 ± 1.97	21.02 ± 1.74	21.36 ± 1.57
BMI (kg/m2)		24.51 ± 1.79	25.23 ± 1.58	24.88 ± 1.66	24.67 ± 1.71
History of exercise (%)	Yes	82%			
	No	18%			
History of smoking (%)	Yes	17%			
	No	83%			
History of drinking (%)	Yes	72%			
	No	28%			
History of Chinese learning (%)	Yes	68%			
	No	32%			

### Procedures

In this study, the parameters of PA and the degree of Chinese learning effort were measured by scales based on various indicators. During the test, all indicators were collected and distributed by SoJum Software (Tencent Holdings Ltd.). Prior to collection, research staff members personally explained the purpose and method of the survey to the respondents, and if they had any questions, they explained them to the respondents face-to-face, and then completed the form with the consent of the counselor, homeroom teacher, or classroom teacher, and with the consent of the respondent. The sale was completed anonymously, without personal data such as ID number or student number, excluding invalid answers, short time (less than 200 seconds), 10 consecutive options with the same and high homogeneity of the questionnaire.

In terms of controlling experimental error, to ensure that there is no potential risk of bias in the results, three measures were used in this study. Firstly, the most internationally recognized and widely used scales were selected in order to obtain good and reliable results in terms of reliability. Secondly, in order to prevent bias in the results due to factors such as the psychological state or lifestyle of the participants, the present study can reduce social approval bias and response fatigue effects by using multiple time points, anonymous completion and response time monitoring during the data collection phase, as well as detecting response consistency by setting up control questions (e.g., reverse questions). Finally, given the possibility of large systematic bias in a single cross-sectional survey, this study conducted three consecutive follow-ups of the same cohort of participants, several months apart, and screened for and excluded anomalous data at the end (anomalous data included: extreme values; too high or too low consistency; contradictory reverse questions; abnormal response times; low internal consistency; and logical self-contradiction).

### Measurements

#### Physical activity level survey.

Physical activity was measured using the short version of International Physical Activity Questionnaire (IPAQ) [20]. IPAQ assigned metabolic equivalent (MET) corresponding to different intensities of PA by asking the study subjects about the frequency of the week (d/wk) and the time of day (min/d) for that intensity. The MET was 3.3 for walking, 4.0 for MPA, and 8.0 for VPA. The PA level for each intensity and the total PA level were calculated separately according to the formula.

In this study, AB was compared with HSK in individuals with varying levels of PA only in the initial survey. This was done to analyze the differences in the various indicators produced by individuals with differing PA habits. Physical activity levels were categorized and grouped into 3 levels according to the criteria recommended by the IPAQ working group [21]: (1) High level PA group (HG). VPA time ≥ 3d per week and total MET ≥ 1500, or PA time ≥ 7d per week and total MET ≥ 3000; (2) Moderate level PA group (MG). Weekly VPA time ≥ 3d and daily PA time ≥20 min, or weekly MPA ≥ 5d and daily PA time ≥30 min, or weekly PA intervals ≥ 5d and total MET ≥ 600; (3) Low level PA group (LG), with PA levels insufficient for the above criteria. The Cronbach α is 0.772 in the study.

#### Aggressive behavior assessment.

AB was measured using the Buss-Perry Aggression Questionnaire (BPAQ), developed by Buss & Perry in 1992 [22]. In the field of psychological research, the BPAQ was the first AB to be used to assess individuals. According to Bushman et al. in 1991, the scale appeared in the Social Science Citation Index a whopping 242 times between 1960 and 1989 [23]. Later, in 1992, Buss and Perry improved the scale again by removing and reintroducing some questions. Because the scale contains sub-dimensions that cover all aspects of AB in a comprehensive way, it has been widely used internationally by various organizations and research teams to assess individuals’ AB in everyday life. The scale consists of 30 questions on a 5-point Likert scale with five dimensions, including physical aggression, verbal aggression, anger, hostility and self-directed aggression, and is scored on a 5-point scale from 1–5. The corresponding response items are ‘not true’, ‘somewhat true’, ‘half true’, ‘mostly true’ and ‘completely true’. and ‘fully meets’. The sum of the sub-indicator scores of each dimension makes up the total score of the scale. In this study, the retest reliability of the scale was 0.84 and the Cronbach’s alpha was α = 0.902.

#### Second language learning achievement.

Hanyu Shuiping Kaoshi (HSK) is the most widely recognized international standardized test of Chinese language proficiency for non-native speakers [24]. Designed and developed by the Sino-Foreign Language Exchange and Cooperation Centre of the Chinese Ministry of Education and the Confucius Institute Headquarters, the HSK classifies learners’ Chinese language skills into “three levels and nine grades” based on their final scores. The assessment criteria include: four basic linguistic elements, namely syllables, Chinese characters, vocabulary and grammar, which form the “four-dimensional benchmark”; three assessment dimensions, namely verbal communication ability, topic and task content, and quantitative linguistic indicators; and five linguistic skills, namely listening, speaking, reading, writing and translating, to accurately calibrate the learner’s Chinese language proficiency. In this study, all subjects were assessed on their Chinese language performance using HSK practice test papers three times each from 2022 to 2023, and the results of each time were included in the final T1, T2 and T3 outcome indicators based on the chronological order.

#### Cross-lagged panel model construction.

The CLPM is constructed based on the results of the 3 measurements by taking 3 longitudinal follow-up measurements of all participants at the following points: T1: September 2022, T2: December 2022 and T3: June 2023. Specifically, the 3 measurements of IPAQ will be used as the main variable and BPAQ will be used as a mediator variable to observe the positive or negative predictive effect of PA on HSK, which is the main dependent variable. The results obtained on three occasions were tested for consistency according to Andersen’s method and the PA, AB and Chinese L2 learning efficacy of the international students were tested for gender and age equivalence by baseline equivalence, load equivalence and intercept equivalence in that order [25]. In this study, Model-1 is the baseline equivalent model, Model-2 is the load equivalent model and Model-3 is the intercept equivalent model.

The results of this study were collected on three separate occasions in September 2022, January 2023 and June 2023. Of these, the first collection (T1) had 100% compliance with no sample loss. The second collection (T2) had a compliance of 95.74% with a loss of 41 samples. The third collection (T3) had a compliance of 89.83% and a loss of 57 samples. The researchers also reported that no risky events occurred during the entire experiment.

### Statistical analysis

In this study, we mainly used comparison of differences, correlation analysis and CLPM to analyze the different indicators. First, SPSS 21.0 was used to test the normality of PA, AB and HSK one by one, and descriptive statistics were performed after determining the types of data. Since the main variables in this study were classified as low, medium and high levels of PA, the test of variance was performed using Martin’s Analysis of Variance (ANOVA) and P, F, partial η² and R2 were reported [26]. Bartlett’s test of sphericity, for data that did not meet the assumptions, the Greenhouse-Geisser method was used to correct the data [27]. To explore the correlation and effect size between variables, Pearson’s test was used to analyze the results and r and P values were reported. After screening the correlation results, valid variables were included in the CLPM by AMOS 24.0 for fitting to explore the path characteristics between variables. After standardizing each result, the CLPM test and confidence interval estimation were performed using model-6 in the SPSS process canonical macro plug-in. The significance of each path was then tested using the bootstrap method [28]. Finally, the Bonferroni test was used for post hoc testing of all indicators, with a significant threshold of P < 0.05.

## Results

### Effects of physical activity levels on Chinese L2 learning and aggressive behavior

**Fig 1:**
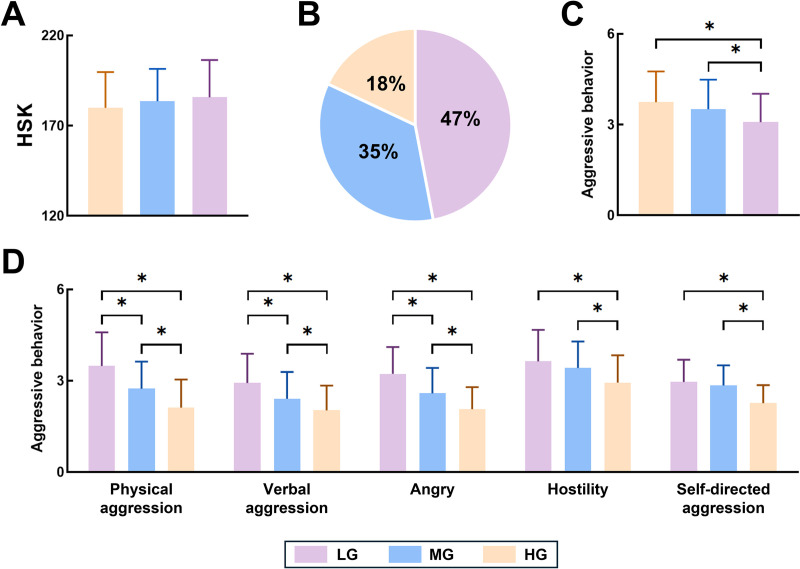
Difference in various variables with different PA level. Note: A: Levels of HSK scores of the three groups of participants. B: Population proportions of the three groups of participants. C: Levels of aggressive behaviors of the three groups of participants. D: Levels of each sub-dimension characterizing aggressive behaviors of the three groups of participants.

The analyses showed that there was a main effect of PA on both HSK and AB. However, ANOVA results showed that there were differences in the effects of different levels of PA on all three. Although the HSK scores of the three groups of subjects were not statistically significantly different, it can be seen through the intuitive parameters that HG > MG > LG. In terms of AB, the level of PA significantly introduced individual differences in physical aggression, verbal aggression, and angry, both the higher the level of PA, the three scores the lower the scores of the three (F = 4.38, P <  0.01, η² = 0.034; F = 3.24, P < 0.01, η² = 0.041; F = 2.37, P < 0.01, η² = 0.050). Furthermore, although the differences in aggressive behavior, hostility, and self-directed aggression between LG and MG were not significant, HG scored significantly lower than the above two groups (F = 1.93, P = 0.039, η² = 0.052; F = 1.58, P = 0.045, η² = 0.05; F = 2.04, P = 0.031, η² = 0.04). The above results also imply that regular PA has an enhanced effect on HSK scores and decreases the level of AB expression. And this effect increases step by step with the level of PA. Detailed results are shown in Table 2 and Fig 1.

**Table 2:** Characterization and difference in various variables with different PA level.

PA Level	N (%)	HSK	Aggressive behavior					
			Aggressive behavior	Physical aggression	Verbal aggression	Angry	Hostility	Self-directed aggression
LG	453 (47%)	179.92 ± 19.76	3.74 ± 1.02	3.49 ± 1.10	2.93 ± 0.96	3.23 ± 0.88	3.64 ± 1.03	2.96 ± 0.73
MG	337 (35%)	183.56 ± 17.95	3.51 ± 0.98	2.74 ± 0.89	2.41 ± 0.88	2.59 ± 0.83	3.42 ± 0.87	2.85 ± 0.66
HG	174 (18%)	185.71 ± 20.66	3.08 ± 0.94	2.12 ± 0.92	2.03 ± 0.81	2.07 ± 0.72	2.94 ± 0.90	2.27 ± 0.59
F			1.93*	4.38**	3.24**	2.37**	1.58*	2.04*
Partial η²			0.04	0.03	0.04	0.04	0.05	0.04
R^2^			0.05	0.04	0.04	0.05	0.06	0.05

Note: PA: Physical activity, HSK: Hanyu Shuiping Kaoshi test, HG: High level PA group, MG: Moderate level PA group, LG: Low level PA group,

* = P < 0.05,

** = P < 0.01, The result is retained to two decimal places only.

### Correlation between physical activity and aggressive behavior in process of Chinese L2 learning

**Fig 2:**
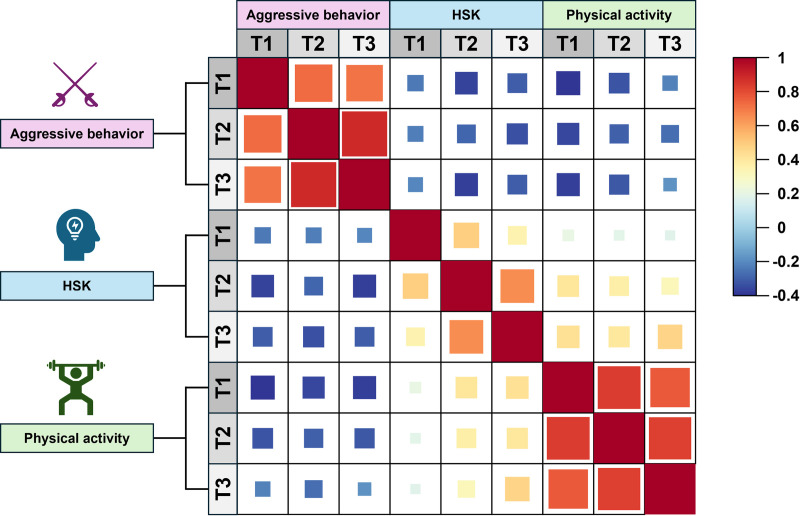
Correlation heatmap between physical activity and individual variables.

The correlation results showed that PA was correlated with HSK and AB by Pearson correlation test. In terms of cross-sectional comparisons, AB was significantly negatively correlated with both HSK and PA (r = −0.41, P < 0.01; r = −0.44, P < 0.01), and HSK scores were positively correlated with PA (r = 0.45, P < 0.01). In terms of longitudinal comparisons, AB showed a gradual increase in its own positive correlation over time (T1-T3) (r = 0.72, r = 0.88, P < 0.01), and a gradual decreasing trend in its negative correlation with PA (r = −0.29, r = −0.44, P < 0.01), but there was no significant change in the degree of negative correlation with HSK (r = −0.28, r = − 0.35, P < 0.01). In the case of HSK, the degree of its own positive correlation decreased somewhat over time (r = 0.47, r = 0.33, P < 0.01) and did not change significantly from the positive correlation with PA (r = 0.15, r = 0.18, P < 0.01). With respect to PA, its internal positive correlation gradually decreased over time (r = 0.83, r = 0.76, P < 0.01). This result also suggests that PA has a positive predictive effect on Chinese L2 learning efficacy and that this effect is relatively stable. Whereas AB has a negative effect on Chinese L2 learning, it can be gradually alleviated by PA intervention. Also, the main variables and all the indirect variables in this study are significantly correlated, thus further CLPM test can be conducted [29]. For detailed information, see Table 3, Fig 2.

**Table 3:** Correlation results between PA and different table variables.

Variable		Aggressive behavior			HSK			Physical activity		
		T1	T2	T3	T1	T2	T3	T1	T2	T3
Aggressive behavior	T1									
	T2	0.72								
	T3	0.7	0.88							
HSK	T1	−0.28	−0.27	−0.25						
	T2	−0.41	−0.33	−0.42	0.47					
	T3	−0.35	−0.38	−0.35	0.33	0.64				
Physical activity	T1	−0.44	−0.4	−0.42	0.15	0.3	0.41			
	T2	−0.37	−0.34	−0.36	0.16	0.35	0.38	0.83		
	T3	−0.29	−0.31	−0.33	0.18	0.39	0.45	0.76	0.82	

Note: HSK: Hanyu Shuiping Kaoshi test, All P-values were less than 0.01 after testing.

### Constructing the cross-lagged panel model based on physical activity with Chinese L2 learning and aggressive behavior

To test model measurement consistency, the study was conducted in 3 steps (baseline equivalence, loadings equivalence, and intercept equivalence) to test the equivalence of subjects’ PA, AB, and HSK, with each of the three metrics across gender and age. The results showed that in terms of equivalence of measures across gender and age, AB and PA satisfied loadings consistency and intercept consistency, and HSK satisfied age loadings and intercept consistency. For gender, there were differences in HSK loadings and intercepts, but no statistically significant differences were found between the 2 adjacent time points (∆CF1 < 0.01), so the model fit index was good. In summary, the measurement items, latent factor meanings, and measurement reference points of subjects’ AB, PA, and HSK scores showed high consistency across gender and age, and the measurements were comparable across gender and age.

Also, Harman’s one-way test was used to test the data for common method bias [30]. A total of 3 tests were conducted, with a total of 17 factors with eigenvalues greater than 1 for the 1st measurement and a maximum factor variance explained of 23.92% (<40%). The 2nd measurement had 12 factors with eigenvalues greater than 1, and the maximum factor variance explained was 31.03% (<40%). The third measurement had 11 factors with eigenvalues greater than 1, and the maximum factor variance explained was 33.29% (<40%). None of the three measurements had serious common method bias problems. For details, see Table 4.

**Table 4:** Cross-lagged panel model consistency test based on physical activity, aggressive behavior and Chinese language learning.

Group	Model	Physical activity						Aggressive behavior						Chinese language learning					
		χ2	df	P	RMSEA	TLI	CFI	χ2	df	P	RMSEA	TLI	CFI	χ2	df	P	RMSEA	TLI	CFI
Gender	Model-1	12.81	2	<0.01	0.09	0.96	0.98	35.26	2	<0.01	0.18	0.92	0.96	5.73	2	0.06	0.06	0.94	0.99
	Model-2	16.02	4	<0.01	0.07	0.99	0.98	36.15	4	<0.01	0.13	0.95	0.96	7.88	4	0.08	0.05	0.96	0.99
	Model-3	16.48	6	<0.01	0.06	0.99	0.98	36.03	6	<0.01	0.1	0.96	0.96	9.32	6	0.11	0.04	0.98	0.99
Age	Model-1	95.04	29	<0.01	0.06	0.96	0.9	96.87	29	<0.01	0.07	0.97	0.92	64.56	29	<0.01	0.05	0.91	0.9
	Model-2	98.58	31	<0.05	0.07	0.96	0.95	97.14	31	<0.01	0.07	0.97	0.92	72.48	31	<0.01	0.05	0.92	0.9
	Model-3	100.79	33	<0.05	0.06	0.97	0.96	98.2	33	<0.01	0.06	0.97	0.92	75.31	33	<0.01	0.05	0.92	0.9

Note: Model-1 is the baseline equivalence model (BEM), Model-2 is the load equivalence model (LEM), Model-3 is the hierarchical linear model (HLM).

**Fig 3:**
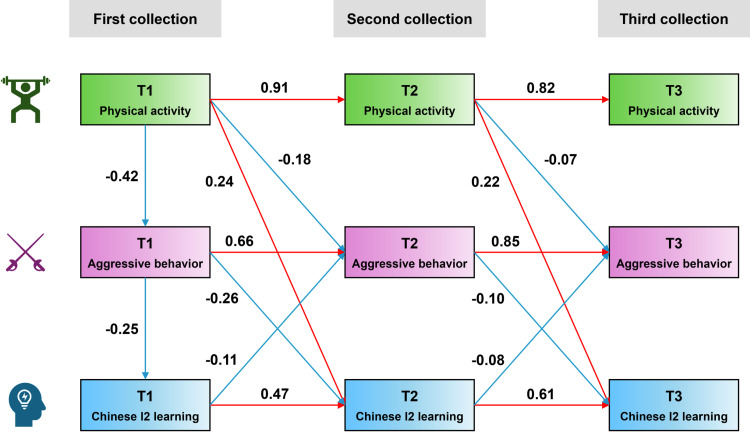
Cross-lagged panel model based on physical activity, aggressive behavior and Chinese language learning.

The results of the CLPM construction showed an overall good model fit for PA, AB, and HSK with χ2/df = 8.90, CFI = 0.97, NFI = 0.94, RFI = 0.88, IFI = 0.97, TLI = 0.90, and RMSEA = 0.12. In particular, T1-PA positively affected T2-HSK scores (β = 0.24), negatively affects T2-AB (β = −0.18). T1-AB negatively affected T2-HSK scores (β = −0.26). T1-HSK scores negatively affected T2-AB (β = −0.11). T2-PA negatively influenced T3-AB (β = −0.07) and positively influenced T3-HSK scores (β = 0.22). T2-AB negatively affected T3-HSK scores (β = −0.10). T2-HSK scores negatively influenced T3-AB (β = −0.08) (all p-values <0.05). For details, see Fig 3.

Meanwhile, in this study, multiple sets of structural equation modelling were used to establish the baseline model (M0) and the qualifying model (cross-lagged paths M1 for boys and girls, as well as the structural intercept M2, structural covariance M3, and structural residuals M4, etc.) and to compare the models in order to examine whether there are any gender differences in the cross-lagged relationships of the variables. The results showed that the models M1 to M4 were well fitted and the differences from the baseline model (CFI = 0.94, TLI = 0.87, RMSEA = 0.12, 90%CI=[0.10, 0.14], P < 0.01) were not statistically significant (∆CFI < 0.05, P > 0.05), i.e., the triad of AB, PA, and HSK scores of all the participants were not statistically significant in the cross-lagged relationship the gender difference was not statistically significant. See Table 5.

**Table 5:** Comparison of the fit of multiple cluster analyses of the cross-lagged panel model.

Model	CMIN	df	P	CMIN/DF	NFI	RFI	IFI	TLI	CFI	RMSEA (95% CI)
Model-0	179.62	33	<0.01	5.69	0.95	0.85	0.96	0.87	0.94	0.12 (0.10, 0.14)
Model-1	190.18	50	<0.01	3.77	0.95	0.88	0.95	0.9	0.95	0.09 (0.08, 0.11)
Model-2	207.64	61	<0.01	3.34	0.92	0.91	0.94	0.92	0.95	0.08 (0.07, 0.10)
Model-3	209.05	62	<0.01	3.29	0.92	0.9	0.95	0.93	0.95	0.09 (0.08, 0.12)
Model-4	247.48	72	<0.01	3.6	0.93	0.88	0.94	0.93	0.94	0.06 (0.05, 0.08)

## Discussion

With the gradual increase of China’s international influence, the number of international students coming to China from all over the world is also gradually increasing. For international students coming to China, learning and using Chinese language is very important in their daily life. At the same time, Chinese language is one of the compulsory courses for every international student. Due to the complexity of its characters and pronunciation, it is not easy to learn Chinese well. For this reason, many language learners refer to Chinese as the ‘devil’s language’. For many students, chronic underachievement is likely to lead to various levels of psychological problems, of which AB is one of the most representative behaviors. AB is one of the most common behaviors that not only impairs self-esteem, self-control and self-efficacy, but also poses a very serious safety risk to others. Research has shown that PA, as a non-pharmacological intervention, can not only effectively improve individual cognitive functions, including language learning and working memory, but also effectively inhibit the occurrence of AB. However, is the effect stable over time and is there a psychological pathway between PA, AB and Chinese language learning? Based on the above questions, this study examines the efficacy of PA in improving Chinese language learning with AB by comparing the effects of different levels of PA on AB and HSK and constructing a CLPM.

The first finding of this study was that there was a beneficial effect of PA on HSK and AB, and that this effect became more pronounced as PA levels increased. In this study, the proportion of people with low PA levels was the largest, while the proportion of people with high PA levels did not exceed one fifth. Therefore, it can be concluded that most new students have a habit of daily exercise or regular PA, but the intensity of the exercise is not high. On the other hand, the higher the level of PA, the higher the HSK scores of the participants and, consequently, the lower the level of AB. Therefore, it shows that PA helps to improve the Chinese learning efficiency of international students and reduce the mental problem behavior. At this point, hypothesis (I) can be confirmed and established. The results also support the findings of previous studies. Similar to the present study, Kormos & Siidetkoon found in a cross-sectional survey that the more frequent the daily exercise, the greater the working memory and cognitive flexibility in second language learning [31]. They are also more motivated and spend more time studying than non-exercisers [32]. In addition, Yashima’s long-term follow-up study, which lasted more than five years, found that in purposeful non-native language environments, individuals’ communication styles undergo a transition from physical behavior to verbal communication. In this process, activities such as PA, especially team sports such as soccer, basketball, or baseball, are more likely to harmonize the ideal L2 self and the ought-to L2 self in the L2 Motivational Self System (L2MSS) and intrinsically transform the second language self into a part of life [33]. Martin argues that most learners’ motivation comes mainly from successful learning experiences in the early stages of language learning, such as collaborative discussions, competitions or sports activities, and is related to the influence of learning context factors such as teachers, classmates and textbooks [34]. On the other hand, for AB, Liu’s screening of three universities in western China found that the effect size of PA on AB was greater than −0.11, and also increased life satisfaction and quality of life [35]. More similarly, to explore the dose characteristics of PA, Yu and Zhuang also divided PA into subgroups according to level. They found that AB decreased with PA level, while the mediating effects of self-control and self-efficacy were 7.28%, 12.26% and 27.70%, respectively [13,36]. Unlike the above studies, although the improvement effects of PA on L2 learning efficacy and aggressive behavior were confirmed, the present study was the first to include all three in CLPM from the perspective of longitudinal screening, and constructed a more stable and reciprocal mental pathway model. Therefore, combining the above results, the present study concluded that more than 3 days of VPA or more than 7 hours of MPA per week is the best form of practice to improve Chinese L2 learning efficacy and alleviate aggressive behavior of international students coming to China.

The facilitatory effect of PA and second language learning versus the inhibitory function of AB can be explained from the perspective of neural mechanisms. From the perspective of the structural basis of the brain, there is evidence that long-term aerobic exercise has a facilitative effect not only on hippocampal volume and neurons, but especially in the adult dentate gyrus [37]. It also enhances the functional connectivity of the Prefrontal Cortex (PFC) and Corpus Callosum [38], modulating blood flow velocity in the middle cerebral and glucose metabolism activity and improving its energy supply environment while increasing its volume and gray matter density and the information integration capacity of the left and right hemispheres [39,40]. Both are involved in verbal cognition and impulse control, and can enhance an individual’s working memory and cognitive flexibility. These abilities involve behaviors such as verbal expression, logical organization, and coping responses. On the other hand, from the perspective of emotion regulation, amygdala and striatum play an important role in the processing of emotions, stress reactions and rewarding behaviors [41]. And it has been shown that regular PA modulates amygdala activity to reduce reactivity to negative stimuli and promotes neuroplasticity in this region [42]. Not only that, but PA also improves striatum activity to modulate the activity of the dopamine pathway. This mechanism is crucial for addiction and reward dependence [43]. From a neurotransmitter perspective, firstly, there is a large body of evidence to show that PA can effectively promote the release of dopamine (DA), serotonin (5-HT), and endorphins [44]. All three not only play a dominant role in emotion regulation and reward mechanisms, but also in psychiatric disorders, including Major Depressive Disorder (MDD) and bipolar disorder (BD) [45]. Specifically, in the nucleus accumbent (NAc) and dorsal striatum regions, PA improved DA signaling by upregulating Dopamine D2 Receptor (D2DR) and Dopaminergic transporter (DAT) [46]. Specifically, PA mediates the addictive mechanisms of individuals during second language learning, thereby improving the learners’ LEARNING EXPERIENCE and increasing the dominance of L2MSS. On the other hand, PA also enhances tryptophan hydroxylase and 5-HT1A receptor ligands activity by promoting the synthesis of 5-HT neurons in the raphe nuclei [47]. Thus, serotonin reuptake transporter (SERT) is inhibited to achieve an increase in an individual’s endearment degree and time spent in the second language, and this effect increases in a stepwise manner with increasing PA levels [48]. In addition, chronically high levels of PA stimulate the secretion of Endocannabinoids, Acetylcholine (Ach) and Gamma-Aminobutyric Acid (GABA) along with other inhibitory transmitters, and increase the expression of α2-adrenoceptor [49,50], N-Methyl-D-Aspartate receptor, α-amino-3-hydroxy-5-methyl-4-isoxazolepropionic acid receptor [51,52], and metabotropic glutamate receptor to stimulate brain regions related to language brain regions and cortical activation associated with language learning and learning control through the LC-NE system [53].

The second finding of this study is that PA positively predicts HSK and negatively predicts AB among incoming international students. In this study, the level of PA among incoming international students is negatively correlated with HSK scores and negatively correlated with AB. Meanwhile, the CLPM test showed that there were interactions between the three, and the effect sizes of the psychological pathways were all highly significant. Although there were no gender differences in the model for this study, consistent with previous studies, boys have higher mean water than girls in all two dimensions of PA and AB. In terms of academic performance, female students have higher scores than male students. The results of the present study suggest gender parity in the longitudinal dynamic associations of international students coming to China. This may be due to the following reasons: among the international students included in the study, there is a relatively even distribution between males and females, making it difficult to observe gender differences. At the same time, the main participants have a similar background in terms of age and educational level, which may also lead to insignificant gender differences. So far, hypothesis (II) can be accepted. Using structural equation modelling (SEM), Adrià found that there was a positive correlation between weekly PA levels and both academic performance and executive functioning in the subjects. Students with high levels of PA were found to have higher GPA, test making, foreign language academic achievement, BMI and CRF than those with medium and low levels of PA [54]. In addition, Akira used aerobic fitness (AF) as a mediator to examine the effects of PA on English learning, family structure and motivation among 608 Japanese middle school students. It was found that there was a direct effect of PA on all three indicators, and the magnitude of the mediating effect of AF was more than 30% [55]. Similar to the present study, Liu also analyzed Chinese students’ English academic efficacy using the L2MSS. It was found that PA positively predicted students’ competence in L2 word picture, vocabulary discrimination, sentence semantic judgement, and listening discrimination in L2 learning, while increasing participants’ ideal L2 self and ought-to L2 self levels [56]. On the other hand, Yuanyuan included AB as a mediator variable in the study and found that PA not only reduced AB, but also effectively inhibited the subjects’ Internet addiction, and the value of the indirect effect was −0.346, accounting for 24% of the total effect [57]. While Du similarly explored the relationship of the pathway and found that the amount of mediating effect of AB was more than 30%, and that the phenomenon was more prevalent among male students than females [58]. In contrast, the present study not only categorized PAs into LG, MG and HG based on level, but also used the more comprehensive IPAQ. Although this may have led to non-standardized comparisons of results across studies, the present study included a much larger sample size and therefore the results were more reliable. Thus, the present study builds on previous research and confirms through longitudinal data that PA increases path stability in second language learning by inhibiting AB.

The above pathways can still be explained by psychological mechanisms, and compensation theory and self-determination theory are good illustrations of what happens to AB when there is academic underachievement. In particular, compensation theory suggests that when an individual has a chronically low level of self-acceptance, he or she will compensate by adopting alternative behaviors and strategies. At the same time, these behaviors may be extreme [59]. Self-determination theory suggests that an individual’s basic motivation comes from the psychological need for autonomy, competence and relatedness. From the perspective of international students coming to China, the high degree of unfamiliarity and language barriers may lead to a loss of self-efficacy due to the immediate change from a familiar native language environment to an unfamiliar area beyond their control. Compensatory psychology also arises and individuals may use AB to counteract expressions of stress or anxiety [60]. In terms of PA, prolonged engagement in sport, especially in group programmers, significantly increases an individual’s self-identity and short-term decision-making capacity, which in turn increases the individual’s cognitive flexibility in executive functioning [60]. In the process of learning Chinese, learners are exposed to various difficulties and stresses in the early stages due to the complexity of tones, rhythms, strokes, etc. Good second language learners need a higher level of stress tolerance and self-regulation. A good second language learner needs a higher level of stress tolerance and self-regulation. This is where people with PA habits sometimes show their strengths. They have a clear goal and a high degree of spontaneity when they engage in PA. As a result, this mental path continues throughout the second language learning process and is manifested in a greater sense of purpose and a higher level of self-determination in long-term PA than in non-persons. This is why Erich Fromm, a famous psychologist, regarded PA as ‘an anesthetic for the maintenance of my self-cognitive and social relationships’ [61]. On the other hand, Substitute Reinforcement Theory explains that when the reward for a behavior is inadequate, individuals will seek an alternative behavior to obtain a similar reward. AB usually provides immediate positive reinforcement when individuals feel failure and anxiety. However, PA, which triggers intracranial transmitter activity, has positive reinforcing properties of its own and can therefore be used as a substitute for AB to some extent [62]. At the same time, long-term PA itself improves posture and further enhances an individual’s overall sense of self (including self-control, self-esteem and self-efficacy) and executive function (inhibition, working memory and cognitive flexibility) [63,64]. For this reason, PA is often cited by the WHO or AHA as the best way to improve mental health and academic performance.

In summary, for international students coming to China, PA can not only serve as an effective approach to enhance Chinese L2 learning efficacy and inhibit AB, but also predict both to some extent by assessing an individual’s daily PA habits. Therefore, based on the findings, this study suggests that language learners from non-Chinese regions can achieve the goal of enhancing their learning efficacy through regular PA in the process of learning Chinese. For schools, organizers can incorporate PA-related activities or competitions into the Chinese language teaching process. For details, see Galal Walker’s Performed Culture Approach [65] and Harold Palmer & A.S. Hornby’s Situational Language Teaching (SLT) [66]. On this basis, learners can be encouraged to maximize the level and intensity of PA within acceptable limits, in line with Principle of Appropriate Load and Principle of Motivation In Sport [67,68]. During this process, single or staged long term exercise programmers can be evaluated and adjusted by RPE, HRmax and MET to achieve optimal PA load levels [69–71].

### Limitations and suggestions for future research

This study compares the differences in L2MSS, learning experience, and intended effort in the process of Chinese language learning among international students with different levels of PA, and explores the correlations and chain relationships among the different indicators. The aim is to improve the process of Chinese language learning and help international students and non-Chinese speaking learners enhance the efficacy of their second language learning. Consequently, a substantial cross-sectional sample of college students from eight colleges and universities in Beijing was included in the study, and the discrepancies in the outcome indicators of diverse aspects of language learning influenced by low, medium, and high PA levels were analyzed, with PA level serving as the primary variable. Nevertheless, this study is not without its limitations.

Firstly, although four indicators were included in this study to facilitate comparison between populations, the experimental design was a cross-sectional survey with no longitudinal follow-up. It should be noted, however, that the experimental design was a cross-sectional survey, and that no longitudinal follow-up study was conducted. In examining previous large sample surveys, it becomes evident that certain studies have employed a screening process utilizing the NHANES, SEER, and CHARLS databases, standardizing the indicators through the application of the Weighted Mean Difference (WMD) methodology, and subsequently conducting sequential comparisons. This approach has the advantage of expanding the sample size while allowing for the analysis of the parameters of the longitudinal intervention within the study. Nevertheless, this approach may result in a loss of the originality of the original research. In lieu of searching previous databases, the present study included and compared a cross-sectional sample of data over a period of approximately one year. It is therefore the intention of this study to highlight its originality, although it must be acknowledged that the results may be open to question due to certain limitations.

Secondly, the study may be affected by the type of research, there is a certain experimental error. This is due to the fact that research through the scale type is realized based on a single cross-sectional survey, so there may be some human or irresistible factors interfering in the process. In this study, such factors include psychological status, lifestyle and academic performance. Therefore, in order to ensure that there was no potential risk of bias in the results, three measures were used in this study. Firstly, the most internationally recognized and widely used scales were selected to provide good and reliable results in terms of reliability. Secondly, to prevent bias due to participants’ psychological conditions or lifestyles, this study used multiple time points, anonymous completion and response time monitoring to reduce social approval bias and response fatigue effects during the data collection phase, as well as control questions (e.g., reverse questions) to test for consistency of responses. Finally, given the potential for large systematic bias in a single cross-sectional survey, this study conducted three consecutive follow-up visits several months apart with the same participants, and screened and excluded outlier data at the end.

While the issues cannot be overlooked, this study endeavored to circumvent the heterogeneity of the results caused by the experimental design through CLPM and other methods within the confines of the limited experimental conditions. Furthermore, future studies will incorporate additional factors, including longitudinal outcomes, physical activity capacity, and gender differences, in order to gain a more comprehensive understanding of the core aspects of the study. Considering the current findings, it is evident that the reliability and rigor of the study will continue to be enhanced on an ongoing basis. To provide an effective exercise prescription and theoretical reference for the international development of Chinese language and linguistic psychology.

## Conclusion

(1) Since the results of ANOVA and correlation showed a significant effect of PA on both HSK and AB, it was hypothesized that PA may help to improve Chinese L2 learning efficacy and reduce AB. (2) The results showed that higher levels of PA were associated with higher HSK scores and lower AB. Therefore, it was inferred that high levels of PA may have the most significant improvement effect on both. (3) The results of CLPM showed that PA had a positive predictive effect on Chinese L2 learning and a negative predictive effect on AB in all three follow-ups. Therefore, it was hypothesized that daily development of PA habits might be an effective approach to enhance Chinese L2 learning efficacy and inhibit AB.
